# Ultraviolet-induced fluorescence dermoscopy in the diagnosis of scabies: The “ball sign”

**DOI:** 10.1016/j.jdcr.2026.01.016

**Published:** 2026-01-21

**Authors:** Varun H, Adarshlata Singh, Bhushan Madke, Ambika Kondalkar, Bhakti Sarda

**Affiliations:** Department of Dermatology, Venereology and Leprosy, Datta Meghe Institute of Higher Education and Research, Jawaharlal Nehru Medical College, Wardha, Maharashtra, India

**Keywords:** ball sign, delta wing jet sign, dermoscopy, scabies, ultraviolet-induced fluorescence dermoscopy, UVFD

## Case description

A 35-year-old male agricultural worker presented with a 2-week history of generalized pruritus with marked nocturnal exacerbation. His wife and adolescent son reported similar symptoms. Pruritus predominantly involved the interdigital spaces, axillae, and groin. There was no history of fever, scaling, discharge, or bleeding.

**Fig 1:**
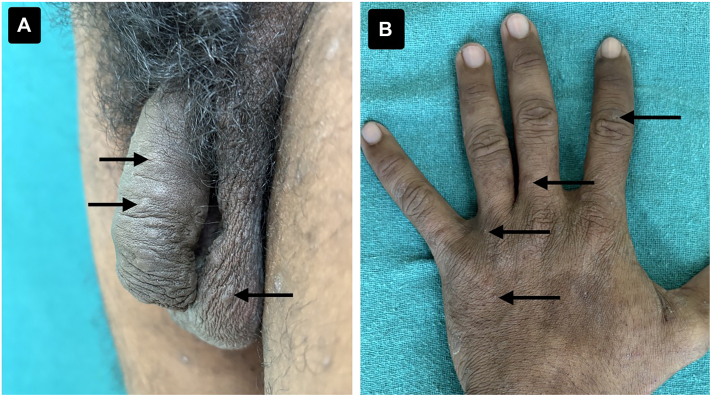
**A,** Clinical image showing multiple well-defined papules and burrows over the penile and scrotal skin (*black arrows*). **B,** Clinical image of the left dorsum of the hand demonstrating multiple ill-defined papules over the interdigital spaces (*black arrows*).

Cutaneous examination revealed multiple ill-defined papules with healed excoriations. Burrows were noted over the interdigital spaces and genital skin (Fig 1).

Dermoscopy of interdigital papules demonstrated ill-defined S- and C-shaped gray-white curvilinear tracts. The classic ‘delta-wing jet’ sign was poorly visualized. Dermoscopic burrow ink test (D-BIT)enhanced visualization of the burrows and revealed a translucent mite at the terminal end.

**Fig 2:**
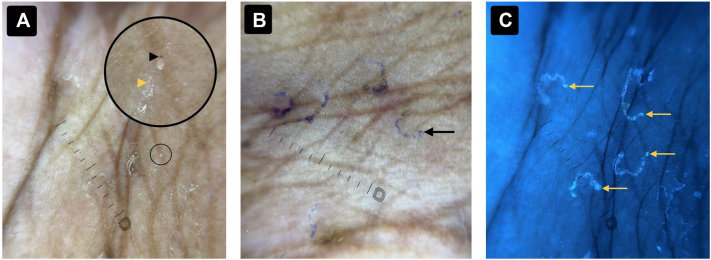
**A,** Dermoscopic image (DermLite DL5 coupled with iPhone 16 camera) showing multiple ill-defined scabietic burrows with the classical ‘delta-wing jet with contrails’ appearance. The *black arrowhead* indicates the ‘delta-wing jet’ or ‘triangle sign’ (mite head), while the *yellow arrowhead* marks the ‘contrail’ (burrow behind the mite). The larger circle highlights the magnified view of the smaller circled area. **B,** D-BIT image of the same region showing multiple burrows filled with ink, with a scabies mite visible within the burrow (*black arrow*). **C,** UVFD image (DermLite Wood-Mode, 365 nm, coupled with iPhone 16 camera) demonstrating well-defined fluorescing scabietic burrows with the ‘ball sign’ (scabies mite) in each burrow (*yellow arrows*). *D-BIT*, Dermoscopic burrow ink test; *UVFD*, Ultraviolet-induced fluorescence dermoscopy.

Ultraviolet-induced fluorescence dermoscopy (UVFD), performed prior to D-BIT, demonstrated bright blue fluorescence of the burrows and a round blue-green fluorescent structure at the burrow tip, consistent with a live scabies mite—termed the “ball sign” (Fig 2). To validate this finding, a skin scraping was performed from the fluorescent area, confirming the presence of live, motile *Sarcoptes scabiei* mites and eggs. These findings confirmed the diagnosis of scabies.

The patient was treated with oral ivermectin (200 μg/kg) administered on an empty stomach, repeated after 1 week. Topical 5% permethrin cream was applied overnight from the neck down following a scrub bath, nail trimming, and subungual cleansing, and repeated after 1 week. Topical 2% fusidic acid was prescribed for excoriations, and oral antihistamines were given for symptomatic relief. All household contacts were treated simultaneously, with concurrent environmental decontamination of potentially infested fomites, including clothing, blankets, and bed linens.

**Question: Which dermoscopic finding under ultraviolet-induced fluorescence dermoscopy is most suggestive of a *live* scabies mite?**

**A.** Delta-wing jet sign
**B.** Triangle sign
**C.** Serpiginous curvilinear tract
**D.** Ball sign
**E.** Ink-filled burrow

## Discussion

Correct answer: D. Ball sign.

Scabies is caused by *Sarcoptes scabiei* var. *hominis* and is traditionally diagnosed based on clinical features such as nocturnal pruritus, involvement of the circle of Hebra, and identification of burrows or mites. Dermoscopy improves diagnostic accuracy by revealing characteristic burrows and mite-related signs, including the triangle sign and delta-wing jet sign. However, these features may be subtle or difficult to visualize, particularly in excoriated or inflamed skin.^1^^,^^2^

UVFD uses 365-nm ultraviolet light to enhance the visualization of skin structures based on fluorescence. In scabies, keratinous debris lining the burrow fluoresces bright blue due to interaction with UV light. Importantly, the body of a live mite emits a distinct round blue-green fluorescence, referred to as the **“ball sign.”** This sign provides a rapid and reliable method for identifying active infestations and may be easier to detect than conventional dermoscopic signs or ink-based techniques.^3^, ^4^, ^5^

Compared with the burrow ink test, UVFD is faster, nonmessy, and avoids excessive staining that can obscure burrow morphology. Incorporating UVFD into routine dermoscopic evaluation can improve diagnostic confidence, particularly in atypical or early presentations of scabies.^3^, ^4^, ^5^

## Conflict of interest

None disclosed.
